# Quackery in New York

**Published:** 1846-08

**Authors:** 


5. *Quackery in New York.*—To the Editor of the Boston Medical and Surgical Journal:

Sir,—For the first time finding myself in the great emporium of everything, ycelpt the London of America, I have thought it might interest your professional readers somewhat, if I should furnish a few notes of my gleanings here in the way of quackery. From all I can see here during a brief sojurn, I should think that the population consists of two classes about equally divided, one half being employed in *making and vending* physic, and the other half in *swallowing* it; but my *penchant* is with the former moiety, among whom I have been making a tour of inspection. The better to effect my purpose I have doffed the doctor, and turned invalid, counterfeiting as you will see, all manner of diseases, and amusing myself by visiting the quacks, and asking questions like a veritable yankee, as I am.

Soon after my arrival, I read a flaming editorial in the papers concerning a certain dentist, who, tired of the slow profits of tooth pulling, announces himself a curer of consumption, having been cured himself. You may be sure that I hastened to see this prodigy, and putting on a woe-be-gone face, obtained an interview. He is a very pale and plausible dentist, I assure you, wholly disinterested and vastly religious, as a man surely ought to be with one foot in the grave, for such a *cure* as his I would not covet, since it gave me the horrors to look at him, and especially to hear his sepulchral voice, but little above a whisper, though he tells of his wonderful cure. All I learned from him was, that he thought I had the consumption, or would be likely to have it, if I did not catch the asthma, which he said was a certain "preventative." He showed me his remedy in the shape of a tube, exactly like those used in Boston and elsewhere by deaf persons, though instead of placing one end to the ear, it is applied to the lips; and he showed me how to breath through the tube by inhaling and expelling the air, which he says affords *exercise* to the lungs, and thus cures the consumption by producing a kind of artificial asthma. He showed me a pamphlet which he benevolently gives away, and a book which he sells along with the tube for five dollars, to those able to buy it, half price to ministers, and, it is said, he gives tubes gratis to the poor. The book is mainly a reprint of the old work of Ramage, of London, entitled "Consumption Curable," which was shown up at the time in the British and Foreign Medical Review, and never before deemed worth re-publication, until this effort to revive his tube in America, after it has become a stale joke in England as the relic of the mountebank St. John Long, from whom Ramage took his cue.

As I was altogether incog., I listened with great gusto to this dentist doctor's cure, for which it seems he went to London, together with an account of the celebrated hospital of Dr. Ramage, under royal patronage, which he described to me as one of the most important public institutions in Great Britain. And he told me of the wonderful cures he had made since his return. I found he was a thorough paced homæopathist, and did not depend upon the tube alone in any case, but advised those who used it, to take the little sugar pellets of Hahnemann, and he boasted of the patronage of that school of physicians in the city, who, it seems, recognize him as a worthy coadjutor.

I need scarcely add that in our conversation he betrayed an utter ignorance of the pathology of consumption, blundering in every attempt to describe or discriminate cases, so that

I left him in amazement that any editor should so far forget himself as to admit into his columns an eulogy upon so illiterate a pretender; but I suppose it is all paid for under cover of advertisements.

I had gone but a little way from his door, before I met an old physician and friend to whom I related my rencontre with this rival of the faculty; from whom I learned that hundreds of these tubes have been bought by the dupes of this folly, and that instances of rapid fatality are known to the profession, resulting from the effort to exercise tuberculous lungs with this villainous tube. The profits of the trade, however, exceed those derived from pulling teeth.

My next visitation was paid to a celebrated advertising quack, who cures all incurable diseases by a combination of homœopathic medicines prescribed by a *sleeping partner* in the person of a lady, who, when her eyes are closed by mesmeric passes, can look into the great cavities of the body, examine minutely the several internal organs, detect the nature and seat of the malady, and direct the preparation of the infallible remedy in every case. The learned doctor has such confidence in his female associate in practice that he does not presume to give an opinion without consulting her ladyship, taking care always to mesmerize her into the somnambulic state, for the reason that she knows nothing at all when awake, but is no sooner put to sleep than she discourses like an oracle upon pathology and therapeutics; whereupon the doctor having received his *fee* for his sleeping partner's advice, is prepared for *another fee* to furnish her prescriptions to the patients. Having learned this state of facts, I retired, not being willing to wait for my turn among so many patients as I found ready to precede me, so that this "craft has great gains," and the twain are driving a profitable trade.

I now thought I would look after the galvanic tribe of quacks who are innumerable here. Electricity, galvanism, and magnetism, separately and combined, are remedial agents greatly in vogue at present among quacks. There are some who use the galvanic battery in the usual way for all cases indiscriminately; while there are others who have magnetic plaster for the outside of the body, with magnetic pills for the inside, by which they have a perpetual current of electro-magnetic fluid flowing with as much certainty and regularity as Prof. Morse's telegraph, provided the patient continues to wear the plaster and take the pills. But all these are mere pigmies compared with the celebrated professors and doctors who vend galvanic rings, bracelets and belts, together with magnetic fluids, and I contented myself with calling on the most celebrated of these. I put his rings on every finger and thumb, with his bracelets on my arms and legs, and his belt about my body, offering to buy them all *for my complaints*, provided I could feel that they had any effect upon my nerves. But though his "fluid" was seduously applied, I had no more evidence of the generation of galvanic influence, than though the rings had been made of wood, the bracelets of hair, and the belt of leather. He assured me that many persons were shocked to the ends of their fingers and toes by applying a single galvanic ring, and he showed me certificates of numerous cures of frightful disease which had been thus wrought. Of course he professed to be astonished at the failure upon my person, and wondered how I could be so insensible, especially as I assured him I was "nervous." I proposed that he should put on the rings himself, and tell me candidly whether he could feel any galvanism; but he declined the test, alleging that he was satisfied with witnessing their success in others, and he appealed to his profitable trade in the articles as proof of their curative powers not to be gainsayed. I declined making any purchases of the mountebank, and pursued my tour among the other quacks of the city, of which you shall hear in my next.

A PERIPATETIC AND COSMOPOLITE.

